# 

**Published:** 1900-09-15

**Authors:** 


### NOTICE TO CORRESPONDENTS.

All MS., Letters, Books for Review, and other matters intended for the Editor should be addressed The Editor, "The Hospital," The Hospital Building, 28 & 29, Southampton Street, Strand, W.C.

The Editor cannot undertake to return rejected MS., even when accompanied by stamped directed envelope.

**Notice to Advertisers and Subscribers.**

All Advertisements, Orders for Copies of The Hospital, and Business Communications should be addressed to **THE MANAGER (Not to the Editor)**, The Hospital, The Hospital Building, 28 & 29, Southampton Street, Strand, London, W.C.

The Cost of Subscription, post free, is as follows:—Per week, 2½d.; per quarter, 2s. 8d.; per half-year, 5s. 3d.; per annum, 10s. 6d. Foreign:—Per annum, 15s. 2d., payable, in all cases, in advance.

**NOTICE.—Vol. XXVII. of "The Hospital" (October, 1899, to April, 1900), in handsome cloth binding, is now on sale at the Office of this Journal, price 7s. 6d. Cases for binding the Numbers contained in this Volume 1s. 6d. Index to Vol. XXVII., 2½d., post free.**

**The Monthly Part for September is now ready. Price 6d., by post 8d.**

### SCRAPS AND GLEANINGS.

DURING the recent thunderstorm the administration block at the Infectious Diseases Hospital at Brighouse was struck by lightning and a portion of the roof demolished, but fortunately no one was injured.

THE bazaar held in the spring in aid of the Hinckley and District Jubilee Hospital realised £1,272, and the subscriptions amounted to £1,400. The cost of the hospital when completed will be £3,000, so that there is a sum of £400 needed to enable the hospital to be opened free of debt.

THE Sherburn Hospital was founded in 1181 as a hospital for lepers. In 1585 it was incorporated by Queen Elizabeth for a master and thirty brethren, and is still subject to the regulations then adopted, At present this is one of the most richly-endowed charitable institutions in the North of England, its income amounting to several thousands of pounds per annum.

THE Hospital Saturday Fund report that the receipts from the workshops and business houses of London from January to June 16th, 1900, amounted to £5,568 odd, being an increase, as compared with the corresponding period of last year, of £143 odd. The expenditure since January reached £878. October 13th has been fixed for the Hospital Saturday collection this year.

THE Countess of Jersey recently opened a sale of work at Sudbury Halt Girls' Home, Wembley. The proceedings were enlivened by the excellent brass band of the "Arethusa" training ship, which played in the extensive garden of the home, and several novel features were introduced including a procession of children in fancy costumes, arranged by local residents. The girls of the home gave an exhibition of musical drill. The Earl and Countess of Jersey had 600 of the boys and girls of the National Refuges Society to spend the day with them at their beautiful park at Osterley, Isleworth, in accordance with their annual custom.

A LARGE company was was present at the opening of the new isolation ward of the Coventry Hospital on July 12th. The new buildings are at the rear of the hospital, but are connected with the main central corridor on the first floor, the principal entrance being closed with folding doors, and cross ventilated. By this means the new block is easy of access yet practically isolated. The accommodation consists of two wards, capable of taking in five patients at a time. The total cost of the extension, with furniture, is estimated at £1,000, and the committee hope to be enabled to meet this without having to resort to the capital account.

THE visit of the Working Men's Committee of the Belfast Royal Victoria Hospital took place on June 30th, and from the report read by Mr. John Ginty (the secretary of the hospital), it appeared that this committee were doing extremely good work. There had, however, been a considerable decrease in the subscriptions received during the last six months, and a rule had therefore been made that young men earning 20s., and married men earning 25s. per week, were not to be eligible for free treatment in the hospital unless they were regular subscribers. Comparing the report of 20 years ago with the present one, it was noticed that in 1879 the total amount of subscriptions received from the working classes was only £179 odd, while the amount of subscriptions received from the working people during the year ending December 31st, 1899, was over £2,500.

THE Victorian Convalescent Home for Surrey Women, and the Princess Mary Memorial Home of Rest for Poor Women, at Bognor, which were opened early in July by the Duke and Duchess of York, are intended to do for women the same good work as the Seaford Convalescent Home for men. These two Homes have been erected at a cost of very nearly £20,000, the whole of which amount has been supplied by Mr. Max Waechter. The buildings are of a superior description. The Victorian Home is making an official appeal foi additional donations and annual subscriptions.

IT has long been customary in Japan for massage to be largely carried out by blind manipulators, both male and female. With this point in view there have been a number of meetings at the offices here of the Gardner's Trust for the Blind in connection with the initiation and development of an institute for the practice of massage by the blind. These meetings have been attended by representative workers for the blind, and the institute is now taking a definite shape. A representative committee has been nominated, with the power to be added to, the chairman being Sir Henry Power, consulting ophthalmic surgeon to St. Bartholomew's Hospital We understand that almost immediately the committee will be seeking publicity for the scheme. The committee of the contemplated institute for massage by the blind are determined that the work done by the blind in this line shall be at least equal and probably superior to anything offered by the seeing manipulators.

AT the annual meeting of the Hull Hospital for Women and Orthopædics the committee's report showed that the number of in-patients treated was 72 and of out-patients 610. The number of operations, which were mostly of a serious character, was 79, and only one death had resulted. The Ladies' Committee, which had been appointed since last year, had performed some very practical work. As regarded the financial position, the hospital had managed to pay its way, but the list of regular contributors was still extremely small, and it was felt that with a little united effort the number of annual subscribers might easily be doubled.

THE Liebig's Extract of Meat Company are advertising their goods in a novel manner. They state that they have "cast adrift on the high seas" a number of Lemco Messenger Buoys containing a message to whoever finds it, "proclaiming in a novel fashion the intrinsic worth of Lemco, and also a coupon for (1) a free week's holiday at the seaside; or (2) a quarter-pound jar of Lemco; or (3) a cloth-bound cookery book." Messrs Liebig's advise visitors to the seaside to keep a careful look out for these buoys, some of which "are certain to drift up on this shore and be found on the sand at the water's edge within the next few days."

### The Student of Medicine.

### APPOINTMENTS.

J. H. BENNETT, M.R.C.S., L.R.C.P.Lond., appointed Medical Officer for the Fourth District of the Maidstone Union. H. E. Davison, M.B., B.S., B.Hy.Durh., M.R.C.S., L.R.C.P.Lond., appointed Resident Medical Officer to the Newcastle-on-Tyne Dispensary. C. A. Ensor, M.R.C.S., L.R.C.P., appointed Medical Officer of Health for the Tisbury Rural District. D. R. Powell Evans, L.R.C.P.Lond, M.R.C.S.Eng., L.S.A.Lond., appointed Medical Officer for the North Wimbledon District of the Kingston Union. H. G. Harris, M.B., B.S.Durh., M.R.C.S., L.R.C.P.Lond., appointed House Surgeon to the Paddington Green Children's Hospital. A. Hewetson, M.R.C.S.Eng., L.R.C.P.Lond., appointed Assistant House Surgeon to the Huddersfield Infirmary. W. C. Rivers, L.R.C.P.Lond., M.R.C.S.Eng., appointed House Surgeon to the Stamford, Rutland, and General Dispensary. W. Sloss, M.B., Ch.B.Edin., appointed Junior House Surgeon to the Bootle Borough Hospital, Liverpool. A. Stevenson, M.B.Glasg., appointed Junior Assistant Medical Officer to Hawkhead Asylum, Crookston.

**GEORGE HARLEY, F.R.S.,**
**Or, The Life of a London Physician.**
By Mrs. ALEC TWEEDIE.
Demy 8vo., with Portrait, cloth gilt, **16s.**

"The authoress has succeeded, by a judicious combination of her father's notes with her own recollections, in producing a readable and interesting memoir."—*The Times.*

London: THE SCIENTIFIC PRESS, LIMITED, 28 & 29, Southampton Street, Strand, W.C.

### THE NURSE'S REPORT BOOK.

Foolscap 4to., Black cover, **6d.** net, post free; or 12 for **4s. 6d.**, post free.

**Compiled by K. H. (Cert.)**

H. J. Glaisher, 57, Wigmore Street, Cavendish Square, W.

